# Spectral Unmixing of Hyperspectral Remote Sensing Imagery via Preserving the Intrinsic Structure Invariant

**DOI:** 10.3390/s18103528

**Published:** 2018-10-18

**Authors:** Yang Shao, Jinhui Lan, Yuzhen Zhang, Jinlin Zou

**Affiliations:** School of Automation and Electrical Engineering, University of Science and Technology Beijing, Beijing 100083, China; b20170299@xs.ustb.edu.cn (Y.S.); yzhang@ustb.edu.cn (Y.Z.); b20160275@xs.ustb.edu.cn (J.Z.)

**Keywords:** spectral unmixing, hyperspectral imagery, intrinsic structure, local window

## Abstract

Hyperspectral unmixing, which decomposes mixed pixels into endmembers and corresponding abundance maps of endmembers, has obtained much attention in recent decades. Most spectral unmixing algorithms based on non-negative matrix factorization (NMF) do not explore the intrinsic manifold structure of hyperspectral data space. Studies have proven image data is smooth along the intrinsic manifold structure. Thus, this paper explores the intrinsic manifold structure of hyperspectral data space and introduces manifold learning into NMF for spectral unmixing. Firstly, a novel projection equation is employed to model the intrinsic structure of hyperspectral image preserving spectral information and spatial information of hyperspectral image. Then, a graph regularizer which establishes a close link between hyperspectral image and abundance matrix is introduced in the proposed method to keep intrinsic structure invariant in spectral unmixing. In this way, decomposed abundance matrix is able to preserve the true abundance intrinsic structure, which leads to a more desired spectral unmixing performance. At last, the experimental results including the spectral angle distance and the root mean square error on synthetic and real hyperspectral data prove the superiority of the proposed method over the previous methods.

## 1. Introduction

**Figure 1:**
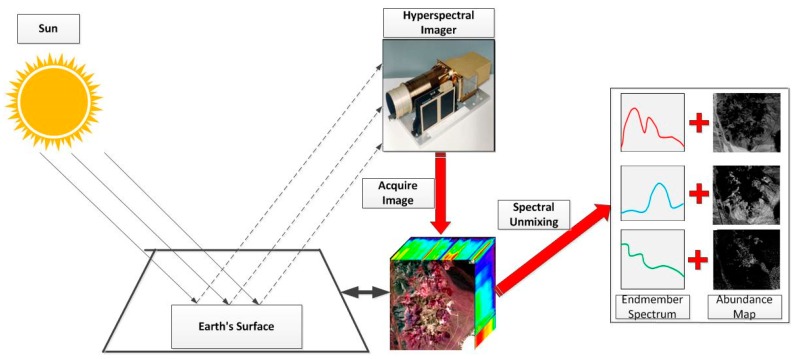
Schematic overview of hyperspectral image acquisition and spectral unmixing.

Airborne and spaceborne hyperspectral remote sensing technology have made remarkable progress in the past two decades. Hyperspectral image is acquired by hyperspectral imager and is composed of pixels formed by tens to hundreds of wavebands in a narrow band (bandwidth less than 10 nm) from 300 nm to 2500 nm. Because of the high spectral resolution of the hyperspectral imagery, it can be used as a reference for identifying the ground object, so the hyperspectral imaging technique shows huge application prospects [1]. The basic unit of the hyperspectral imager that receives the ground signal is the pixel. Each pixel records an electromagnetic signal reflected by surface materials in the spot on the ground corresponding to the (one-pixel) instantaneous field of view (IFOV) of the hyperspectral imager, which is called spectral information. The spot may contain different ground objects. These ground objects have different spectral signals which are the basic components of spectral signal of the pixel. If one pixel contains only one ground object, the pixel is a pure pixel. If one pixel contains multiple ground objects, the pixel is a mixed pixel. Mixed pixels arise for one of two reasons. First, if the spatial resolution of the hyperspectral imager is low enough that adjacent ground objects can jointly occupy a single pixel. Second, mixed pixels appear when distinct materials are combined into a homogeneous mixture [2]. Due to the technical bottleneck in the design and manufacture of hyperspectral imagers, the spatial resolution of hyperspectral data is limited to a certain extent even though super-resolution techniques are utilized [3], so the mixed pixels are common in hyperspectral remote sensing images. To identify the ground objects and their proportions in the mixed pixels is meaningful. Spectral unmixing aims to decompose the spectrum of mixed pixels into a set of constituent spectra, or endmembers, and a set of corresponding fractions, or abundances, which indicate the proportion of endmembers in the pixel [2,4]. Figure 1 shows the schematic overview of hyperspectral image acquisition and spectral unmixing.

The various research communities have proposed numerous methods for spectral unmixing. Based on the assumption that there is at least one pure pixel per endmember in hyperspectral images, many scholars have proposed corresponding algorithms, such as N-finder (N-FINDER) [5], vertex component analysis (VCA) [6], simplex growing algorithm (SGA) [7], and maximum volume by Householder Transformation (MVHT) [8]. In fact, due to the limited spatial resolution of the hyperspectral imager, there are a large number of mixed pixels in the hyperspectral remote sensing images. The resulting value of the spectral unmixing methods based on the assumption of pure pixels will have a large error. Therefore, some scholars have proposed some spectral unmixing methods without adopting pure pixels assumption, such as minimum volume simplex analysis (MVSA) [9], simplex identification via split augmented Lagrangian (SISAL) [10], and dependent component analysis (DECA) [11]. In addition, there are methods that can generate endmembers and abundance information at the same time, such as non-negative matrix factorization (NMF) [12] and minimum volume enclosing simplex (MVES) [13]. In the above methods, because NMF can generate endmember matrix and abundance matrix at the same time and is suitable for the extraction of mixed pixels, the research of NMF theory is the focus of many scholars. NMF theory is applied to spectral unmixing in the literature [14]. However, this method simply performs mathematical operations and lacks clear geographical significance [14]. To apply NMF theory to spectral unmixing, different scholars add different constraints to the standard non-negative matrix factorization objective function, making the mathematical model more in line with the actual geographical significance. They optimized the corresponding mathematical model solving method and achieved a certain spectral unmixing effect [15,16,17,18,19,20,21]. MVC-NMF [18] regards the minimum monomorphic volume formed by endmembers as a constraint. L_1/2_-NMF [20] with sparseness constraints is proposed to obtain the best sparse solution of endmember abundances.

Hyperspectral images are characterized by large amounts of data, high dimensions, and high band correlation. The different bands of hyperspectral images, especially the adjacent bands, are highly correlated, resulting in a certain degree of information redundancy [22]. Especially for hyperspectral classification, the number of bands is not positively correlated with the accuracy. On the contrary, when the number of bands reaches a certain limit, the overall classification accuracy will decrease, resulting in the so-called Hughes phenomenon [23]. It makes sense to reduce the dimensions of hyperspectral images. Manifold learning [24] is a nonlinear method of dimension reduction. Manifold learning (e.g., Laplacian Eigenmap [25]) is to find the low-dimensional manifold in the high-dimensional space and to find the corresponding embedded mapping. Researchers have shown that the image data cannot fill up uniformly the high-dimensional Euclidean space [25]. The image data can be viewed as data sampled from a low dimensional manifold embedded in a higher-dimensional space. These image data smoothly change along the geodesic of the data manifold [25]. All above-mentioned spectral unmixing algorithms only consider the Euclidean structure of the hyperspectral data space. In fact, hyperspectral data are more likely lie on low-dimensional manifold [26]. Inspired by manifold learning, we transform the hyperspectral image data projection into a low-dimensional space to model its intrinsic structure, thereby realizing the hyperspectral image dimension reduction to facilitate spectral unmixing. It is known that hyperspectral image not only contains abundant spectral information of the ground objects, but also includes spatial distribution of the ground objects. Most of the above mentioned methods treat hyperspectral images as a collection of spectral vectors and neglect the possible spatial correlations between pixels. Yet, in the literature [27], a prior of spatial correlations between the different pixels of the hyperspectral image is utilized in spectral unmixing algorithm which leads to improve spectral unmixing accuracy. Weighted non-negative matrix factorization designs appropriate weights integrating the spatial information in local neighborhood to enhance spectral unmixing [28]. Inspired by this, the spatial information of the hyperspectral image is preserved when modeling the intrinsic structure of the hyperspectral image. Endmember spectral variability is an inevitable phenomenon in hyperspectral imaging and is a source of error in spectral unmixing accuracy [29]. A hierarchical weighted sparsity unmixing (HWSU) method improves spectral unmixing accuracy by decreasing the influence of the endmember spectral variability [30]. In modeling the intrinsic structure of hyperspectral image, it is necessary to consider reducing the influence of spectral endmember variability to improve the accuracy of hyperspectral unmixing. Meanwhile, we attempt to establish the close link between hyperspectral image and abundance matrix and construct the graph regularization to preserve intrinsic structure invariant in hyperspectral unmixing. In addition, sparse constraint that reduces the risk of getting stuck in local minima in non-convex minimization computations will be introduced into the proposed method in the paper, which enhances the sparsity of endmember abundances.

Finally, a novel algorithm which can preserve intrinsic structure invariant in hyperspectral unmixing named PISINMF is proposed for hyperspectral unmixing. PISINMF improves two aspects to improve spectral unmixing accuracy. Firstly, L_1/2_ regularizer with regularization parameter defined in the literature [31] is adopted as sparsity constraint for improving the sparsity of endmember abundances. Secondly, the intrinsic structure of hyperspectral image is modeled, which preserves spectral information and spatial information of hyperspectral images. The above mentioned intrinsic structure, as a priori knowledge, is constructed with graph regularization that makes the intrinsic structure of the decomposed abundance information consistent with the intrinsic structure of the true abundance information. PISINMF is experimented on synthetic hyperspectral data and real hyperspectral data to verify its validity.

The rest of the paper is organized as follows: the second part introduces the proposed method of PISNMF, the third part introduces the experimental results and analysis of synthetic data and real data, the fourth part introduces discussion and the fifth part introduces the conclusions.

## 2. The Proposed PISINMF Method

**Figure 2:**
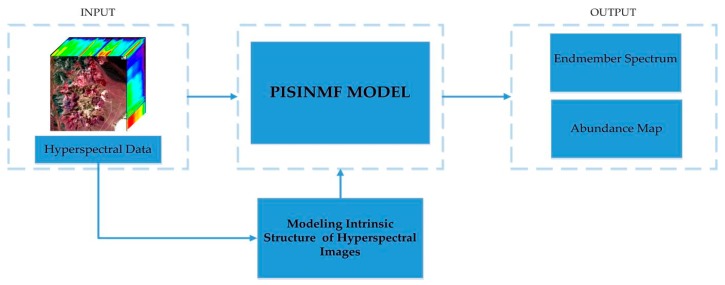
Schematic overview of the proposed method.

Mixed pixels are typically modeled using a linear mixture model or a nonlinear mixture model. As a rule of thumb, the linear mixturemodel is associated with mixtures for which the pixel components are homogeneous surfaces in spatially segregated patterns. On the contrary, the nonlinear mixture model takes the intimate association or interaction with more than one component into account [30]. The linear mixture model is simpler than the nonlinear model, and the linear model’s calculation results meet the accuracy requirements. The linear mixture model can explain the formation of hyperspectral image and conform to the actual statistical laws. In this paper, we will introduce the proposed algorithm model based on the linear mixture model. The schematic overview of the proposed method is shown in Figure 2.

### 2.1. Linear Mixture Model

The linear mixture model is based on the following three assumptions. Firstly, a finite number of endmembers within each IFOV linearly contribute pixel spectral signals according to their coverage percentage (abundance). Secondly, the ground objects are homogeneous surfaces in spatially segregated patterns. Thirdly, the electromagnetic energy of neighboring ground objects within each IFOV does not affect each other [32,33,34].

Based on the above assumptions, the linear mixture model of the *l*-band hyperspectral image containing *n* pixels with *m* endmembers can be expressed as follows:

(1)  X=GA+ε 

where $X=\left[x_{1},\ldots ,x_{n}\right]\in {\mathbb{R}}^{l\times n}$ represents the hyperspectral image ($x_{i}$ represents the *i*th pixel regarded as *l*-band spectral vector, *l* denotes band number, *n* denotes number of hyperspectral image pixels); $G=\left[g_{1},\ldots ,g_{m}\right]\in {\mathbb{R}}^{l\times m}$ represents the endmember matrix ($g_{i}$ represents the *i*th endmember signature); $A=\left[a_{1},\ldots ,a_{n}\right]\in {\mathbb{R}}^{m\times n}$ denotes abundance matrix; ε denotes an error matrix meaning the noise of hyperspectral image. In general, X is a known hyperspectral image. Endmember matrix G and abundance matrix A are the solution targets.

$A_{ij}$ denotes the proportion of the area occupied by the *i*th endmember in the *j*th pixel. According to the linear mixture model assumption, the abundance matrix A needs to satisfy requirements that each element within it is non-negative and the sum over the columns of A are unity.$\;A_{ij}$ obeys the following constraints expressed in Equation (2):

(2) $$\;\begin{array}{c} A_{ij}\geq 0,\forall i,j\\ \sum_{i=1}^{m}A_{ij}=1,j=1,2,\ldots ,n\; \end{array}\;$$

### 2.2. Modeling Intrinsic Structure of Hyperspectral Images

The proposed method transforms the hyperspectral image data projection into a low-dimensional space to model its intrinsic structure preserving spectral information and spatial information of hyperspectral images, thereby realizing the low-dimensional representation of the hyperspectral image to facilitate spectral unmixing.

**Figure 3:**
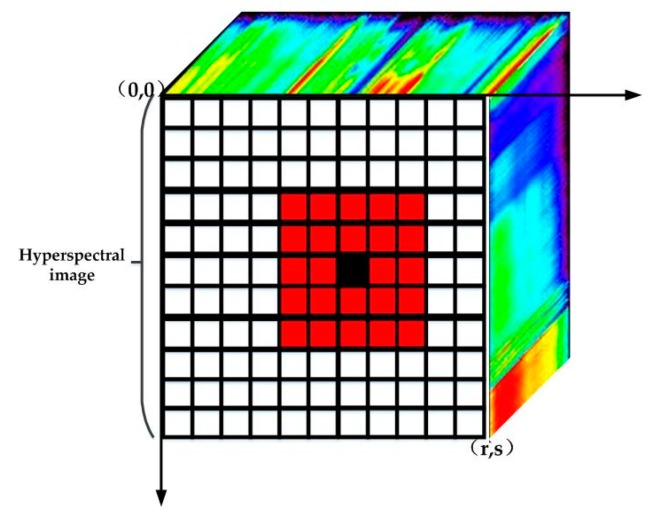
Diagram of the local window of hyperspectral image.

The first law of geography is expressed as “Everything is related to everything else, but near things are more related than distant things” [35]. With that in mind, we divide the hyperspectral image into multiple local windows, considering only the relation between the central pixel and the surrounding pixels in the local window, ignoring the pixels outside the local window. Figure 3 shows the local window of hyperspectral image. Assume that the hyperspectral image with *n* pixels, equals to *r × s* pixels, is arranged on *r* *×* *s* grids. The spatial coordinate of the top left corner grid is (0, 0). The grid spatial coordinate is increased from left to right, from top to bottom until the coordinate of the bottom right corner grid is (*r*,*s*). In Figure 3, a black grid and red grids constitute a local window in which the black grid represents central pixel and red grids represent surrounding pixels. The size of the local window can be expressed by the product of the number of grids at the outermost edge of the local window. For example, the size of the local window in Figure 3 is 5 × 5. $x_{i}\;$and $x_{j}$ are the hyperspectral image pixels which represent spectral signals. Let us consider the case in which pixel $x_{i}$ is the central pixel of a local window. If the pixel $x_{j}$ is a surrounding pixel of pixel $x_{i}$ in the local window, we define j∈N(i). If the pixel $x_{j}$ does not belong to surrounding pixels of pixel $x_{i}$ in the local window, we define j∉N(i).

Then we model the intrinsic structure of the local window of the hyperspectral image. $x_{i}$ and $x_{j}$ are connected by an edge if the pixel $x_{j}$ is a surrounding pixel of pixel $x_{i}$ in the local window. The edges can be weighted by the heat kernel [25]. The heat kernel is expressed as follows:

(3) $$\;K_{\sigma }\left(x_{i},x_{j}\right)=\{\begin{array}{l} e^{-\frac{{\left\|x_{i}-x_{j}\right\|}^{2}}{\sigma }},j\in N\left(i\right)\\ 0,\;j\notin N\left(i\right) \end{array}\;$$

where σ is a scaling parameter of the heat kernel. $x_{i}$,$x_{j}$ are the *i*th and *j*th column vectors of the hyperspectral image X. It is known from Equation (3) that when the pixel $x_{j}$ is in the local window, it is connected to the central pixel $x_{i},$ and the strength of the connection can be weighed by the heat kernel. When the pixel $x_{j}$ is outside the local window, its connection to the central pixel does not exist. This is consistent with the first law of geography. The σ value adapted to different intrinsic structures of local windows is given by the following equation:

(4) $$\;\sigma =\frac{1}{h-1}\underset{j\in N\left(i\right)}{\sum }{\left\|x_{i}-x_{j}\right\|}^{2}\;$$

where *h* is the number of surrounding pixels of the central pixel $x_{i}$ in the local window.

A hyperspectral image not only contains abundant spectral information of ground objects, but also includes spatial correlations between each pixel. Utilization of spatial correlations between pixels will be incorporated in modeling the intrinsic structure of hyperspectral image. Inspired by the above principle, the closer the surrounding pixel $x_{j}$ is to the spatial distance of the central pixel $x_{i}$, the more the surrounding pixel $x_{j}$ contributes to the central pixel $x_{i}$. It is also obvious that the closer the spectral similarity between the surrounding pixel $x_{j}$ and the central pixel $x_{i}$, the more the surrounding pixel $x_{j}$ contributes to the central pixel $x_{i}$. Assume that (*p*, *q*) and (*b*, *c*) are the spatial coordinates of the central pixel $x_{i}$ and the surrounding pixel $x_{j}$, respectively. Then $x_{i}\;$and $x_{j}$ also can be expressed as ***x***(*p*, *q*) and ***x***(*b*, *c*). Accounting for the effects of spectral information and spatial information, how much the surrounding pixel $x_{j}$ contributes to the central pixel $x_{i}$ can be reflected by Equations (5)–(7):

(5) $$\;M_{i,j}=\{\begin{array}{c} \frac{1}{\sqrt[{}]{\mu }\times \nu },j\in N\left(i\right)\\ 0,\;j\notin N\left(i\right) \end{array}\;$$

(6) $$\;\mu =\sqrt[{}]{{\left(p-b\right)}^{2}+{\left(q-c\right)}^{2}}\;$$

(7) $$\;\nu =arccos\left(\frac{\langle x\left(p,q\right),x\left(b,c\right)\rangle }{\left\|x\left(p,q\right)\|\|x\left(b,c\right)\right\|}\right)\;$$

where < ***x***(*p*, *q*), ***x***(*b*, *c*)> denotes the inner product of the two spectra, and ||·|| denotes the vector magnitude. μ value characterizes spatial distance difference between the central pixel $x_{i}$ and the surrounding pixel $x_{j}$. The smaller the value of μ, the closer the surrounding pixel $x_{j}\;$is located with respect to the central pixel $x_{i}\;$and the more contribution pixel $x_{j}$ has to the central pixel $x_{i}$. ν represents the spectral angle distance between $x_{i}$ and $x_{j}.$ Spectral angle distance reflects the difference in the geometric characteristics of the two spectral vectors. If the ν value is smaller, the more similar the geometric feature of the surrounding pixel $x_{j}\;$is to the central pixel $x_{i}$ and the more contribution the pixel $x_{j}$ has to the central pixel $x_{i}$. $M_{i,j}$ is a simplified representation of the degree of the contribution. Equation (5) is convenient to calculate and can reflect the effect of the surrounding pixel $x_{j}$ on the central pixel $x_{i}$. Accounting for the effect of the surrounding pixel $x_{j}$ on the central pixel $x_{i}$, we promote Equations (3)–(8):

(8) $$\;W_{i,j}=\{\begin{array}{c} M_{i,j}*e^{-\frac{{\left\|x_{i}-x_{j}\right\|}^{2}}{\sigma }},j\in N\left(i\right)\\ 0,\;j\notin N\left(i\right) \end{array}\;$$

If the value of $W_{i,j}$ is larger, the surrounding pixel $x_{j}$ is more closely related to the central pixel $x_{i}$. Compared with Equation (3), Equation (8) better reveals spectral geometric differences, spatial distance information, and spectral similarity of pixel pairs in the local window.

**Figure 4:**
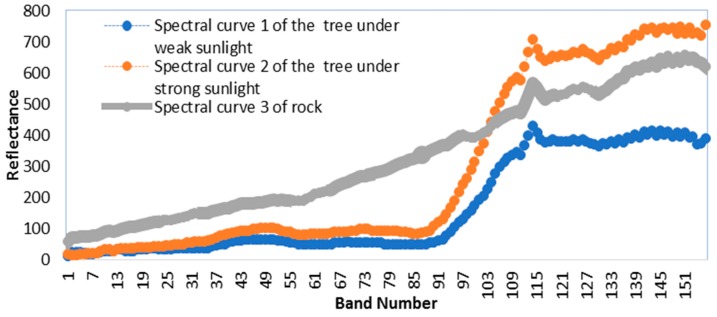
Spectral curve of ground objects.

**Figure 5:**
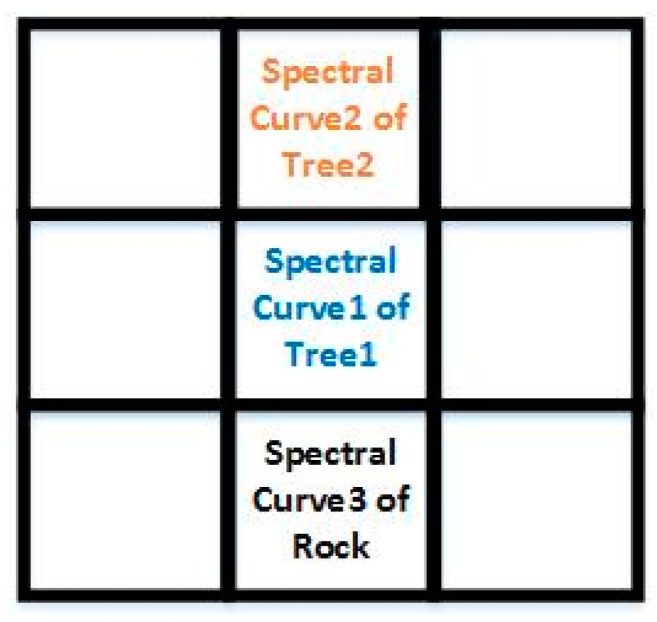
Spatial position of the ground objects of Figure 4.

The spectral information of the pixel reflects the physical and chemical characteristics of the ground objects in the hyperspectral image. However, the spectral curve of the same ground object will change under different environments, which is called endmember spectral variability. Figure 4 shows the spectral curves of trees and rocks. As can be seen from Figure 4, curve 1 of the blue line is the spectral curve of trees under weak sunlight, curve 2 of orange line is the spectral curve of trees under strong sunlight, and curve 3 of gray line is the spectral curve of rocks. Because of the change in the intensity of solar radiation, the spectral curve of the tree mutates. The spatial position of the three ground objects of Figure 4 is as shown in Figure 5. It is assumed that the ground object of the central pixel is tree1 whose spectral information is curve 1. The ground objects of the two surrounding pixels are tree2 and rock whose spectral curves are curve 2 and curve 3, respectively.

We use Equations (3) and (8) to project the relation between the central pixel and the surrounding pixels into the low-dimensional space. The resulting projection values are shown in Table 1. After projecting by Equation (3), projection value of the relation between tree1 and tree2 is relatively equal to projection value of the relation between tree1 and rock. According to the projection results of Equation (3), we think that the effect of the two surrounding pixels on the central pixel is nearly the same, and that the ground objects of the two surrounding pixels are close, which is obviously contrary to the actual situation. After projecting by Equation (8), projection value of the relation between tree1 and tree2 is much larger than projection value of the relation between tree1 and rock. According to the projection results of Equation (8), compared with the surrounding pixel including the curve 3, we determine that the surrounding pixel containing the curve 2 is closer to the central pixel, which is in accordance with the real situation.

Hyperspectral image can be segmented into homogeneous and transition areas [27,28,36]. Pixels in homogeneous areas have large values relatively close to each other [28]. Pixels in transition areas have relatively small values compared with homogeneous pixels [28]. Segmenting the homogeneous and transition areas of hyperspectral images is conductive to improve spectral unmixing accuracy [27,28]. For each central pixel $x_{i}$ with coordinate (*p*, *q*), the whole relations with its all surrounding pixels in the local window can be calculated in the Equation (9). $W_{i,j}$ can be used to calculate the strength of the central pixel $x_{i}$ connection with surrounding pixel $x_{j}$. The whole relations between the central pixel $x_{i}$ with coordinate (*p*, *q*) and its all surrounding pixels in the local window can be denoted as φ(p,q) or φ(i):

(9) $$\;\varphi \left(p,q\right)=\varphi \left(i\right)=\underset{j\in N\left(i\right)}{\sum }W_{i,j}\;$$

**Figure 6:**
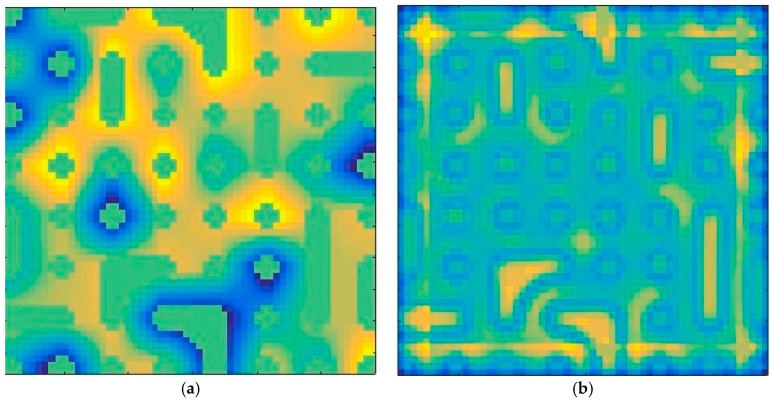
Example of homogeneous and transition areas segmentation in hyperspectral image: (**a**) simulated hyperspectral image; (**b**) the whole relations of all local windows of simulated spectral image.

Given hyperspectral image with *r × s* pixels, the whole relations of all local windows of hyperspectral image can be described as ***R*** matrix in the Equation (10). Figure 6 shows simulated hyperspectral image and whole relations of all local windows of simulated spectral image according to Equation (10). We see that Figure 6b inherits the spatial distribution characteristics of the simulated hyperspectral image. In Figure 6b, yellow represents a larger value and blue represents a smaller value. According to the definition of homogeneous and transition areas, the areas of dark yellow and light yellow are homogeneous areas while the blue areas are transition areas. Therefore, ***R*** matrix can segment the homogeneous and transition areas of hyperspectral image:

(10) $$\;R=\left[\begin{array}{ccc} \varphi \left(1,1\right) & \cdots & \varphi \left(1,s\right)\\ \vdots & \ddots & \vdots \\ \varphi \left(r,1\right) & \ldots & \varphi \left(r,s\right) \end{array}\right]\;$$

Based on the above analysis, we choose Equation (8) as the projection equation, which will project the hyperspectral image into the low dimensional space. We determine each pixel of the hyperspectral image as the central pixel to create a local window with size of 5 × 5. Then we use the projection Equation (8) to project the relation between the central pixel and the surrounding pixels into the low-dimensional space to model the intrinsic structure of hyperspectral images.

### 2.3. PISINMF Algorithm Model

Spectral unmixing is designed to extract endmember spectrum and corresponding abundance from hyperspectral image data, in accordance to the linear mixture model expressed by Equation (1). NMF has been introduced into spectral unmixing, which aims at obtaining endmember matrix G and abundance matrix A to approximately represent the given non-negative matrix X. In order to measure the degree of approximation, the loss function based on the square of the Euclidean distance between X and GA is expressed as:

(11) $$\;\underset{G,A}{\min}f\left(G,A\right)={\left\|X-GA\right\|}_{F}^{2}\;$$

where the operator ${\left\|.\right\|}_{F}$ represents the Frobenius norm.

Unfortunately, because of the non-convexity of NMF, the algorithm has a large number of local minima. In order to reduce the influence of the non-convexity of NMF on spectral unmixing accuracy, sparsity constraints are introduced into standard NMF [14,20]. In [20], L_1/2_ regularizer has been proven to provide the best sparse solution. Therefore, L_1/2_ regularizer is adopted as sparsity constraint for improving the sparsity of endmember abundances. Thus, NMF with sparsity constraints is written as:

(12) $$\;\underset{G,A}{\min}f\left(G,A\right)={\left\|X-GA\right\|}_{F}^{2}+\lambda {\left\|A\right\|}_{1/2}\;$$

where λ is the regularization parameter which weights the contribution of ${\left\|A\right\|}_{1/2}$. ${\left\|A\right\|}_{1/2}$ is defined in the Equation (13):

(13) $$\;{\left\|A\right\|}_{1/2}=\overset{m}{\underset{i=1}{\sum }}\overset{n}{\underset{j=1}{\sum }}A_{ij}^{1/2}\;$$

Motivated by a temperature schedule in the simulated annealing technique, the regularization parameter λ is defined in the Equation (14) to avoid getting stuck in local minima. The regularization parameter λ shows its effectiveness in [31]. Thus, it will be adopted as the regularization parameter that controls the impact of the sparsity measure function ${\left\|A\right\|}_{1/2}$:

(14) $$\;\lambda =\alpha_{0}e^{-\;\frac{t}{\tau }}\;$$

where $\alpha_{0}$ and τ are constants to regularize the impact of sparsity constraints and t is the iteration number in the process of optimization [31].

Given the hyperspectral data ${\left\{x_{i}\right\}}_{i=1}^{n}$, the abundance data ${\left\{a_{i}\right\}}_{i=1}^{n}$, and the endmember matrix ***G***, the following relation exists in Equation (15):

(15) $$\;x_{i}=Ga_{i}\;$$

$x_{i}\in {\mathbb{R}}^{l}$ is an individual pixel of hyperspectral image X. $a_{i}\in {\mathbb{R}}^{m}$ is abundance data corresponding to endmembers. From the perspective of dimensionality reduction, the endmember matrix can be regarded as a new base matrix in the m-dimension space. The hyperspectral image is represented as a linear combination of the new base matrix. $a_{i}$ can be regarded as the representation of $x_{i}$ in m-dimension space. Equation (15) can be further promoted to Equation (16):

(16) $$\;\|x_{i}-x_{j}\|=\|G(a_{i}-a_{j})\|\;$$

We learn from Equation (16) that the more similar $x_{i}$ and $x_{j}$ are, the more similar the abundance of given pixel $x_{i}$ is to the abundance of given pixel $x_{j}$. Therefore, a close link between $x_{i}$ and $a_{i}$ reveals a close link between hyperspectral image and decomposed abundance matrix. Based on the above analysis, once $x_{i}$ and $x_{j}$ are close, the abundance $a_{i}$ of pixel $x_{i}$ and the abundance $a_{j}$ of pixel $x_{j}$ in the m-dimension space are close too. Inspired by the above analysis, a graph regularizer is introduced in PISINMF to keep local structure invariant between hyperspectral image and decomposed abundance matrix. Graph regularizer is written as:

(17) $$\;\overset{n}{\underset{i=1}{\sum }}\underset{j\in N\left(i\right)}{\sum }{\left\|a_{i}-a_{j}\right\|}^{2}W_{i,j}=\overset{n}{\underset{i=1}{\sum }}a_{i}^{T}a_{i}D_{ii}-\overset{n}{\underset{i=1}{\sum }}\underset{j\in N\left(i\right)}{\sum }a_{i}^{T}a_{j}W_{i,j}=\mathrm{Tr}\left(A\left(D-W\right)A^{T}\right)\;$$

where Tr(.) represents the trace of matrix,$\;{\left(\cdot \right)}^{T}$ represents the transpose of matrix, W is weight matrix in which $W_{i,j}$ is element, ***D*** is a diagonal matrix and $D_{ii}=\underset{j\in N\left(i\right)}{\sum }W_{i,j}$.

To make PISINMF have the ability to preserve the intrinsic structure invariant, the regularization term of Equation (17) obtained in the previous section is incorporated into the Equation (12). The cost function of PISINMF is written as:

(18) $$\;\underset{G,A}{\min}f\left(G,A\right)={\left\|X-GA\right\|}_{F}^{2}+\lambda {\left\|A\right\|}_{1/2}+\frac{\mu }{2}\mathrm{Tr}\left(A\left(D-W\right)A^{T}\right)\;$$

where μ≥0 is the regularization parameter of the graph regularizer.

### 2.4. The Update Rules for PISINMF

The projection gradient learning [14] method following the standard gradient learning is adopted to obtain the iterative rule for PISINMF. To make ***G*** and ***A*** non-negative, we use the function max{0,x} to set the negative components to zero while leaving the non-negative components unchanged. Based on the iterative rule, the non-negative matrix ***X*** is decomposed to obtain ***G*** and ***A***. If the result of the *k-*th iteration is ***G***^(*k*)^ and ***A***^(*k*)^, the iterative rule is written as:

(19) $$\;G^{\left(k+1\right)}=\max\left\{0,G^{\left(k\right)}-\alpha^{\left(k\right)}\nabla_{G}f\left(G^{\left(k\right)},A^{\left(k\right)}\right)\right\}\;$$

(20) $$\;A^{\left(k+1\right)}=\max\left\{0,A^{\left(k\right)}-\beta^{\left(k\right)}\nabla_{A}f\left(G^{\left(k\right)},A^{\left(k\right)}\right)\right\}\;$$

where $\alpha^{\left(k\right)}$ and $\beta^{\left(k\right)}$ denote the learning step, $\nabla_{G}f\left(G,A\right)$ and $\nabla_{A}f\left(G,A\right)$ are the first-order derivatives of the function f(G,A) expressed in Equations (21) and (22):

(21) $$\;\nabla_{G}f\left(G,A\right)=2GAA^{T}-2XA^{T}\;$$

(22) $$\;\nabla_{A}f\left(G,A\right)=2\left(G^{T}GA+0.5*\lambda A^{-\frac{1}{2}}+\mu *A*D\right)-2\left(G^{T}X+\mu *A*W\right)\;$$

If α and β are equal to some small positive numbers, the equations are equivalent to conventional gradient descent. When setting $\alpha =G/\left(GAA^{T}\right)\;$ and $\beta =A/G^{T}GA$, the iterative rule of ***G*** and ***A*** could be turned into the multiplicative update rule [27]. The multiplicative rules are written as:

(23) $$\;G\leftarrow G.*\left(XA^{T}\right)/\left(GAA^{T}\right)$$

(24) $$\;A\leftarrow A.*\left(G^{T}X+\mu *A*W\right)./\left(G^{T}GA+0.5*\lambda A^{-\frac{1}{2}}\;+\mu *A*D\right)\;$$

where .∗ and ./ represent the multiplication of elements and the division of elements within the matrix, respectively.

### 2.5. Implementation Issue

The multiplicative rules of PISINMF algorithm model do not consider the sum over the columns of abundance to be unity. To make the results of PISINMF algorithm model more accurate, ***X*** and ***G*** are replaced by $\bar{X}$ and $\bar{G}\;$in Equation (25):

(25) $$\;\bar{X}=\left[\begin{array}{c} X\\ \delta 1_{n}^{T} \end{array}\right]\;\bar{G}=\left[\begin{array}{c} G\\ \delta 1_{m}^{T} \end{array}\right]\;$$

where $1_{n}\left(1_{m}\right)$ is a *n* (*m*)-dimensional column vector of all 1 s. *δ* in Equation (25) is the weight value and can control the influence of the sum over the columns of abundance to be unity on the objective function. In the implementation, a relatively small value of *δ* (i.e., δ≤40) will cause the sum of each column of ***A*** to be much smaller than one. On the contrary, a larger value of *δ* (i.e., δ≥60) will cause the sum of each column of ***A*** to be closer to one, but it reduces the convergence rate. So *δ* is selected as 50 to meet the needs of precision and efficiency in PISINMF model.

In general, the initial value of the endmember matrix ***G*** can be calculated by the endmember extraction algorithm or randomly chosen data. In PISINMF algorithm model, we use the VCA algorithm or other endmember extraction algorithms to set the initial value of endmember matrix ***G***.

The initial value of the abundance matrix ***A*** can be calculated using the least squares method expressed as following Equation (26):

(26) $$\;A={\left(G^{T}G\right)}^{-1}G^{T}X\;$$

To make the initial value of the abundance matrix ***A*** non-negative, we force the matrix ***A*** to be non-negative according to Equation (27):

(27)  A=max(A,0) 

The PISINMF algorithm model sets two stop criteria in the iterative optimization process. One of the stop criteria is the maximum number of iterations, another stop criteria is that a threshold *τ* should be specified for the Equation (28):

(28) $$\;\frac{1}{n}\overset{n}{\underset{i=1}{\sum }}\sqrt{\frac{1}{l}{\left\|X-GA\right\|}_{F}^{2}}\;\leq \;\tau \;$$

### 2.6. The Procedure of PISINMF

The procedure of PISINMF is summarized as follows:

Step 1. Determine the endmember number m; initialize the endmember matrix ***G*** by VCA algorithm or other endmember extraction algorithms for synthetic hyperspectral data and real hyperspectral data; initialize the abundance matrix ***A*** using Equations (26) and (27);
Step 2. Update ***G*** by Equation (23);
Step 3. Replace matrices ***G*** and ***X*** with matrices $\bar{G}$ and $\bar{X}$ according to Equation (25);
Step 4. Update ***A*** by Equation (24);
Step 5. Replace matrices $\bar{G}$ and $\bar{X}$ with matrices ***G*** and ***X***;
Step 6. Repeat step 2–step 5 until any one of the stop criteria is satisfied.

## 3. Experimental Results and Analysis

The paper uses synthetic hyperspectral data and real hyperspectral images to verify and analyze the algorithm model. The experiment is to verify the effectiveness of the PISINMF algorithm and to compare the performance with classical algorithms.

Spectral angle distance (*SAD*) is often used to calculate the degree of approximation between the estimated endmember spectrum and the true endmember spectrum. *SAD* is defined as follows:

(29) $$\;SAD_{i}=arccos\left(\frac{{g_{i}}^{T}\hat{g_{i}}}{\left\|{g_{i}}^{T}\|\|\hat{g_{i}}\right\|}\right)\;$$

where $g_{i}$ is the estimated *i*th endmember signature and $\hat{g_{i}}$ is the *i*th endmember signature. The smaller calculated SAD value, the closer the extracted spectral endmember is to the true spectrum.

The root mean square error (RMSE) of the entire image measures the similarity between the estimated abundance and the true abundance. RMSE is written as:

(30) $$\;\mathrm{RMSE}=\sqrt{\frac{1}{n}\sum_{i=1}^{n}{\left(a_{i}-\hat{a_{i}}\right)}^{2}}\;$$

where $a_{i}$ is a column vector of the estimated abundance A and $\hat{a_{i}}$ is a column vector of the true abundance. If the calculated RMSE value is smaller, the accuracy of the decomposed abundance matrix can be considered to be closer to the true abundance.

The parameters of the PISINMF algorithm model are as follows: (1) the maximum number of iterations is set to 1000 in all the synthetic hyperspectral data and real hyperspectral data experiments; (2) regularization parameter μ in PISINMF algorithm model is experimentally selected between $0.005\;n/m^{2}$ and $0.05\;n/m^{2}$ for all the synthetic hyperspectral data and real hyperspectral data experiments, with which PISINMF algorithm model can reach a satisfactory performance; (3) threshold τ should be specified as 0.001; (4) $\alpha_{0}$ and τ are selected as 0.1 and 25, respectively; (5) The size of the local window is selected as 5 × 5 in PISINMF.

### 3.1. Synthetic Hyperspectral Data

**Figure 7:**
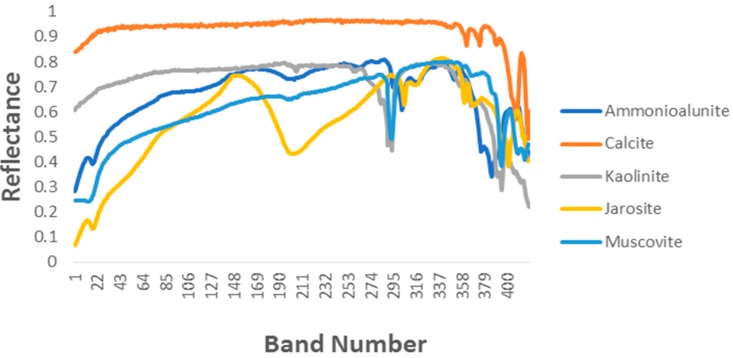
Five endmember signatures extracted from the USGS Spectral Library.

Five endmember signatures (ammonioalunite, calcite, kaolinite, jarosite, muscovite) have been extracted from the USGS Spectral Library [37]. As shown in the Figure 7, the endmember signatures have 420 spectral bands. The five endmember signatures are mixed to form corresponding abundance matrix according to the Dirichlet distribution, ensuring that abundance is non-negative and the sum of each column of the abundance matrix is unity. Mixing coefficients are used in order to make some pixels mixed in higher degree. If the abundance of a pixel is larger than mixing coefficient, the pixel is replaced with a mixture made up of all endmembers of equal abundances. Different experimental environmental conditions, such as different signal-to-noise ratios (SNR), different abundance mixing coefficients, and different spatial dimensions are set to test the unmixing ability of the PISINMF algorithm. In the same environment the unmixing ability of the PISINMF algorithm and other algorithms is compared. The results of the comparison are evaluated using SAD and RMSE standards. To evaluate anti-noise ability, zero-mean white Gaussian noise is added to the synthetic hyperspectral data. SNR is defined as:

(31) $$\;\mathrm{SNR}=10log_{10}\frac{E\left[X^{T}X\right]}{E\left[\varepsilon^{T}\varepsilon \right]}\;$$

where X and ε represent the observation and noise of pixels, respectively. *E*[·] denotes the expectation operator.

Firstly, the synthetic hyperspectral data experiments verify that the PISINMF algorithm has the ability to preserve intrinsic structure invariant. The size of the synthetic hyperspectral data is 30 × 30, the abundance mixing coefficient is 0.8, and the signal-to-noise ratio is 30 db, respectively. PISINMF, L_1/2_-NMF, VCA are used to obtain the estimated abundance from the synthetic hyperspectral data. Then, the estimated abundance and the true abundance are projected onto the low-dimensional space using the Equation (8). In order to compare the ability of three methods (PISINMF, L_1/2_-NMF, VCA) to preserve the intrinsic structure, Equation (32) is used to compare the difference between the projection value of the estimated abundance and the projection value of the true abundance. If the error value is smaller, the ability to preserve the intrinsic structure is stronger:

(32) $$\;\mathrm{NORMp}=\sqrt{\sum_{i=1}^{n}{\left(P_{i}-\hat{P_{i}}\right)}^{2}}\;$$

where $P_{i}$ stands for the projection value of the estimated abundance and $\hat{P_{i}}$ stands for the projection value of the true abundance.

**Figure 8:**
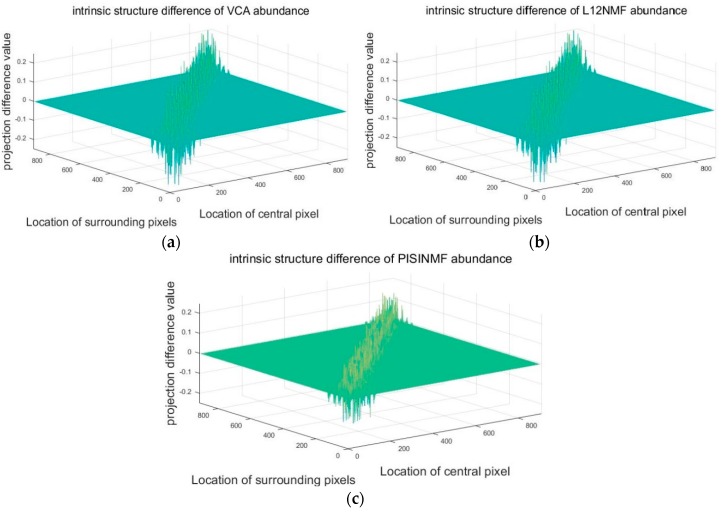
The difference between the intrinsic structure of the true abundance and the intrinsic structure of the estimated abundance (**a**) The difference between the intrinsic structure of the true abundance and the estimated abundance of VCA; (**b**) The difference between the intrinsic structure of the true abundance and the estimated abundance of L_12-_NMF; (**c**) The difference between the intrinsic structure of the true abundance and the estimated abundance of PISINMF.

**Figure 9:**
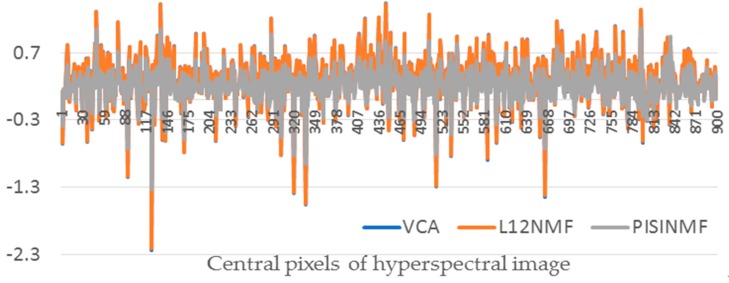
Sum of difference values between central pixels and all surrounding pixels in the local window.

The error values of the three methods are recorded in Table 2. The difference between the intrinsic structure of the true abundance and the intrinsic structure of the estimated abundance is shown in Figure 8. To more visually show the difference, we accumulate the error between the central pixel of Figure 8 and its surrounding pixels in the local window. Sum of difference values based on the central pixels in the local window is shown in Figure 9. In Table 2, NORMp of PISINMF is smaller than the other two methods (L_1/2_-NMF, VCA). As can be seen from Figure 8 and Figure 9, compared to the other two methods (L_1/2_-NMF, VCA), the intrinsic structure of the estimated abundance obtained by PISINMF is closer to the intrinsic structure of true abundance.

**Figure 10:**
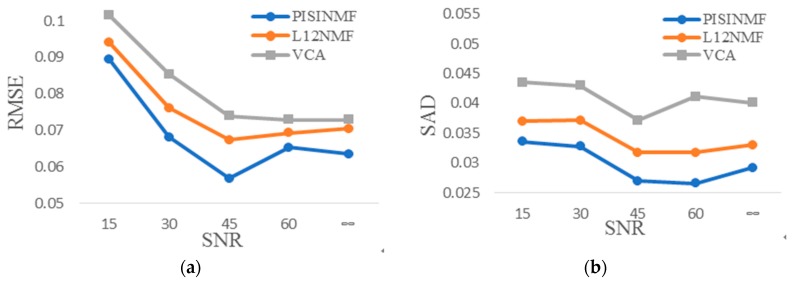
Experimental results with different SNR (**a**) RMSE; (**b**) SAD.

The synthetic hyperspectral data experiments verify the anti-noise ability of the PISINMF algorithm model. We test the anti-noise ability of PISINMF, L_1/2_-NMF and VCA under different SNR conditions. The number of pixels in the synthetic hyperspectral data is 2401, the abundance mixing coefficient is 0.8, and the signal-to-noise ratios are 15, 30, 45, 60, ∞ db, respectively. Performance is evaluated using SAD and RMSE standards. The accuracy of the extracted endmember spectra is evaluated using the average value of SAD, and the accuracy of the obtained abundance is evaluated by RMSE. Table 3 provides SAD values for each method and Table 4 provides RMSE values for each method under different SNR conditions. Figure 10 shows the plots of the experimental results with different SNR. By comparison, PISINMF has smaller RMSE and SAD values than the other two algorithms (L_1/2_-NMF, VCA) under different SNR conditions. Particularly when SNR = 15 dB, the SAD and RMSE values of PISINMF are obviously superior to that of L_1/2_-NMF and VCA.

**Figure 11:**
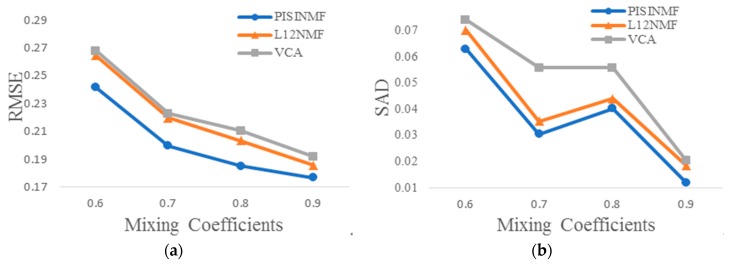
Experimental results with different abundance mixing coefficients: (**a**) RMSE; (**b**) SAD.

Experiments test the PISINMF algorithm’s unmixing ability under different abundance mixing coefficients. Therefore, the synthetic hyperspectral dataset has a signal-to-noise ratio of 35 db, a number of pixels of 2401, and abundance mixing coefficients of 0.6, 0.7, 0.8, and 0.9, respectively. Table 5 provides SAD values for each method and Table 6 provides RMSE values for each method under different abundance mixing coefficients conditions. Figure 11 shows the plots of the experimental results with different abundance mixing coefficients. As can be seen from Figure 11, SAD value and RMSE value of the three methods show a downward trend as abundance mixing coefficients increase. By comparison, RMSE value and SAD value of the PISINMF model algorithm are smaller than those of the other two algorithms (L_1/2_-NMF, VCA) under different abundance mixing coefficients.

**Figure 12:**
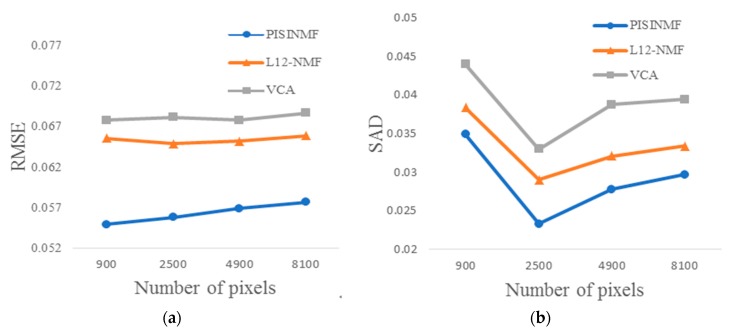
Experimental results with different numbers of pixels in the scene: (**a**) RMSE; (**b**) SAD.

Experiments test the algorithm’s unmixing ability under different spatial dimensions conditions. Spatial dimension here is referred to the number of mixed pixels. The synthetic hyperspectral dataset has a signal-to-noise ratio of 35 db, an abundance mixing coefficient of 0.8, and the number of pixels are set to 900, 2500, 4900, and 8100, respectively. The accuracy of endmember spectra is evaluated using the mean value of SAD and the accuracy of the abundance is evaluated using RMSE. Table 7 provides SAD values for each method and Table 8 provides RMSE values for each method under different spatial dimensions conditions. Figure 12 shows the plots of the experimental results with different numbers of pixels. In Figure 12, we can see that the SAD value and RMSE value of PISINMF show a relatively stable trend with the increase of the number of pixels, indicating that the number of pixels has limited influence on the precision of spectral unmixing. By comparison, PISINMF has smaller RMSE and SAD values than the other two algorithms (L_1/2_-NMF, VCA) under the conditions of different numbers of mixed pixels, indicating that the PISINMF algorithm is suitable for different number of pixels.

### 3.2. Real Hyperspectral Data

**Figure 13:**
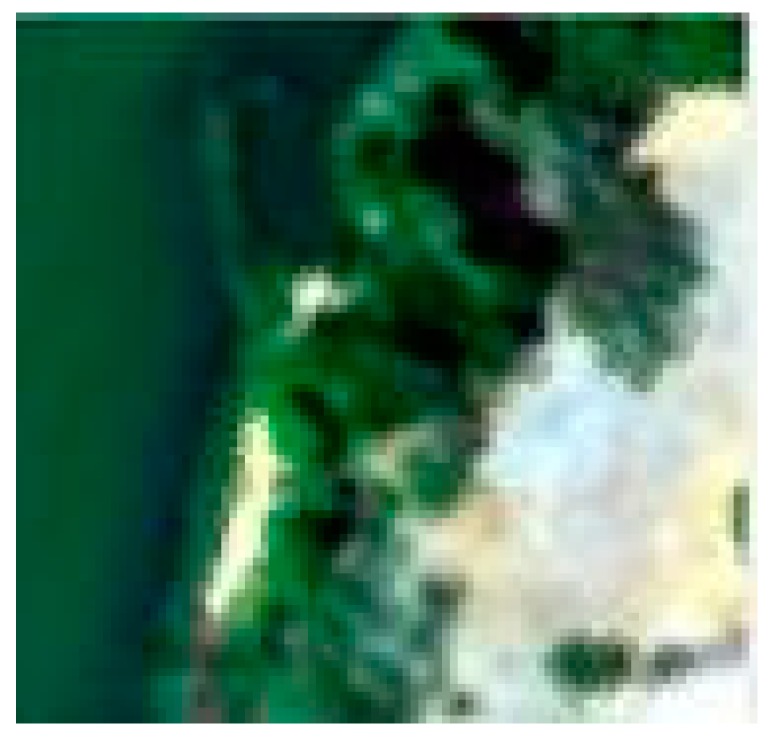
Subimage of Samson hyperspectral dataset.

In this section, there are two real hyperspectral data used to verify the algorithm. The first real hyperspectral dataset is Samson hyperspectral image which covers the spectral range of 400 nm–900 nm with a band width of 3.2 nm. The hyperspectral dataset has been available on the internet [38]. This data is owned by Oregon State University and is provided by WeoGeo. The hyperspectral dataset was acquired by the SAMSON instrument, a push-broom, visible to near IR, hyperspectral sensor. This dataset has been atmospherically corrected using TAFKAA, a hyperspectral atmospheric correction algorithm. This data is in units of remote sensing reflectance. As the original image with 952 × 952 pixels is too large, a subimage with 95 × 95 pixels has been extracted from the original image [39]. A region of 95 × 95 pixels is shown in Figure 13.

By visual observation, the image is mainly covered by water, tree and rock. So the number of the endmembers is selected as 3. The reference endmember spectra are obtained according to the literature [30]. Some pixels corresponding to the three ground objects are randomly selected in Figure 13 by visual observation. The number of pixels selected for each ground object is 30. We calculate the average value of pixels corresponding to each ground object as the reference endmember signature.

**Figure 14:**
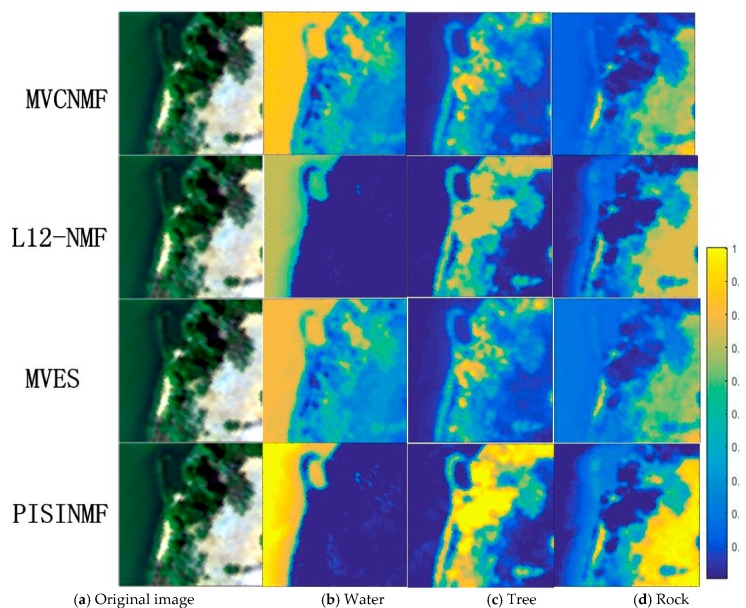
Abundance maps of MVC-NMF, MVES, L_1/2_-NMF, and PISINMF for the Samson dataset: (**a**) Original image; (**b**) Water; (**c**) Tree; (**d**) Rock.

Table 9 provides SAD values for each method (PISINMF, L_1/2_-NMF, MVC-NMF, MVES), and the best result is denoted by bold font. As can be seen from Table 9, PISINMF has the highest number of the best-performance cases. The abundance maps for L_1/2_-NMF, MVC-NMF, MVES and PISINMF are shown in Figure 14.

**Figure 15:**
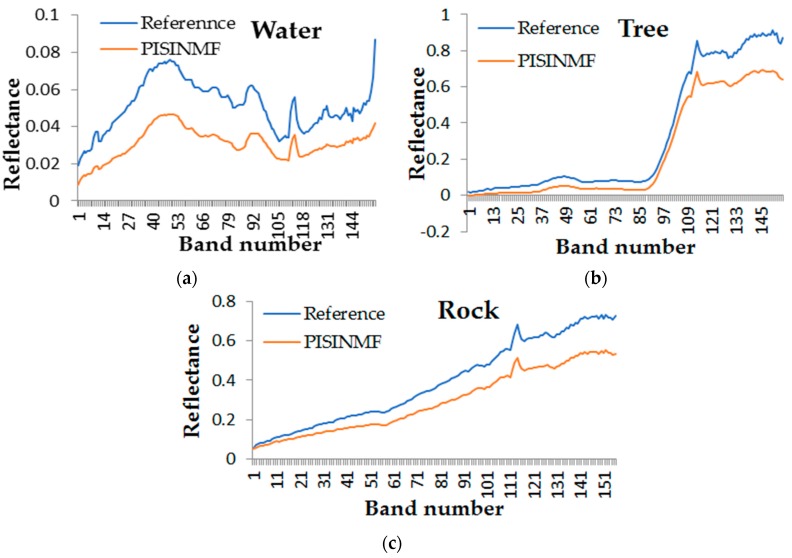
Results on the SAMSON image: Comparison of the reference spectra (blue line) with the endmember spectra extracted by PISINMF (orange line): (**a**) Water; (**b**) Tree; (**c**) Rock.

Figure 15 displays a comparison of the endmember spectra of the PISINMF algorithm with the reference spectra. The color map value of the abundance map is shown in Figure 14. The color of the abundance map corresponds to the value 0–1. The closer the color is to the blue, the closer the abundance value is to 0. This indicates that there is no such ground object distribution in the area. The closer the color is to the yellow, the closer the abundance value is to 1, indicating that more ground objects are distributed in the area. Compared with the abundance maps of other methods, we can see that the abundance map of PISINMF is closer to the distribution of real ground objects. The water, rock and tree abundance maps of the PISINMF algorithm are basically consistent with the original map. However, in the water abundance maps of the MVES and MVCNMF algorithms, some areas of the water abundance map are misjudged. Although the abundance maps of L_1/2_-NMF are similar to PISINMF, the abundance maximum value of PISINMF is relatively close to 1 while the abundance maximum value of L_1/2_-NMF is about 0.7. According to the meaning of the color map of abundance map, the abundance information of PISINMF is closer to the real situation by visual observation.

**Figure 16:**
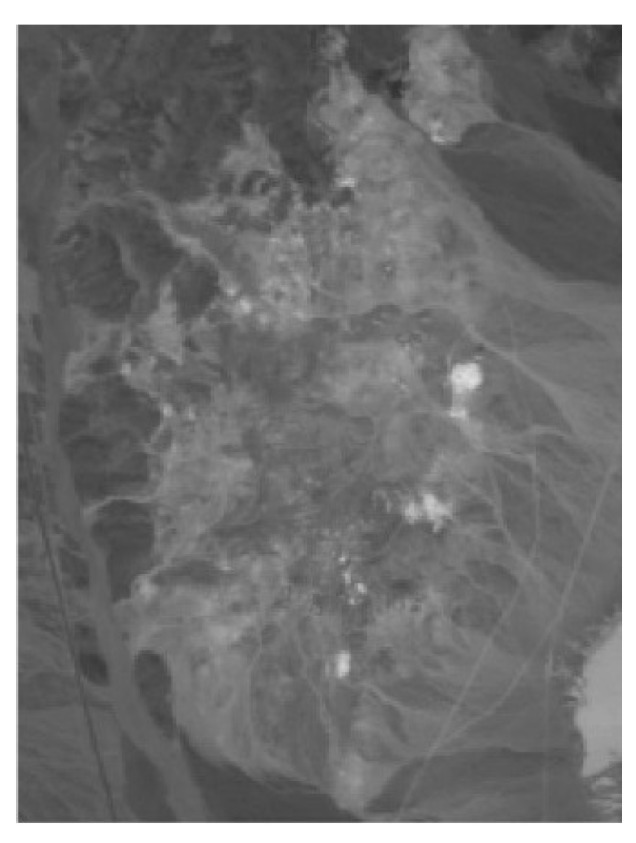
Subimage of AVIRIS Cuprite data (band 80).

The section will analyze the second real hyperspectral data about the Cuprite mineral area in the western part of Nevada, USA, which is a mineralogical site that has been established as a reference site for hyperspectral and other remote sensing instruments [40,41,42]. This image was obtained by an Airborne Visible-Near/Infrared Imaging Spectrometer (AVIRIS) sensor on 19 June 1997. Cuprite data has been widely used to validate spectral unmixing algorithm. The real spectral library of ground objects in this area is available online [37]. Experiments will use the PISINMF algorithm to extract endmembers and abundance maps corresponding to endmembers from the image. The experiment uses the USGS Spectral Library as reference standard and uses SAD to measure the accuracy of the extracted endmember spectra. The subimage of 250 × 190 pixels is taken from the real hyperspectral data for experimentation. The subimage has been atmospherically corrected [6]. Figure 16 shows the 80th band of the subimage. In experiments, the low SNR bands and the water vapor absorption bands (1–6, 104–113, 148–167, and 219–224) have been removed [20]. The number of endmembers in the selected region is estimated to be 12 using the VD method [43] with false alarm probability $P_{f}=10^{-5}$. The endmember signatures extracted by the PISINMF algorithm and other algorithms are compared with the USGS library, and spectral similarities are quantified using the SAD standard. In the results, kaolin is divided into two endmembers due to the endmember spectral variability.

**Figure 17:**
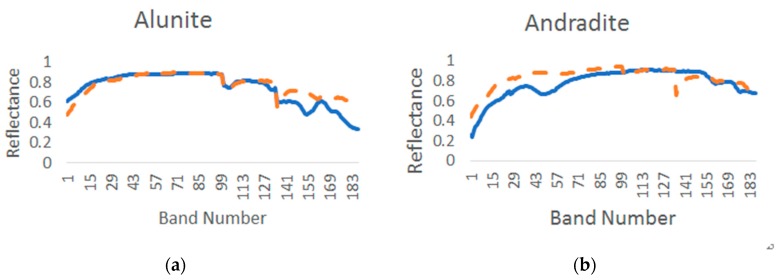

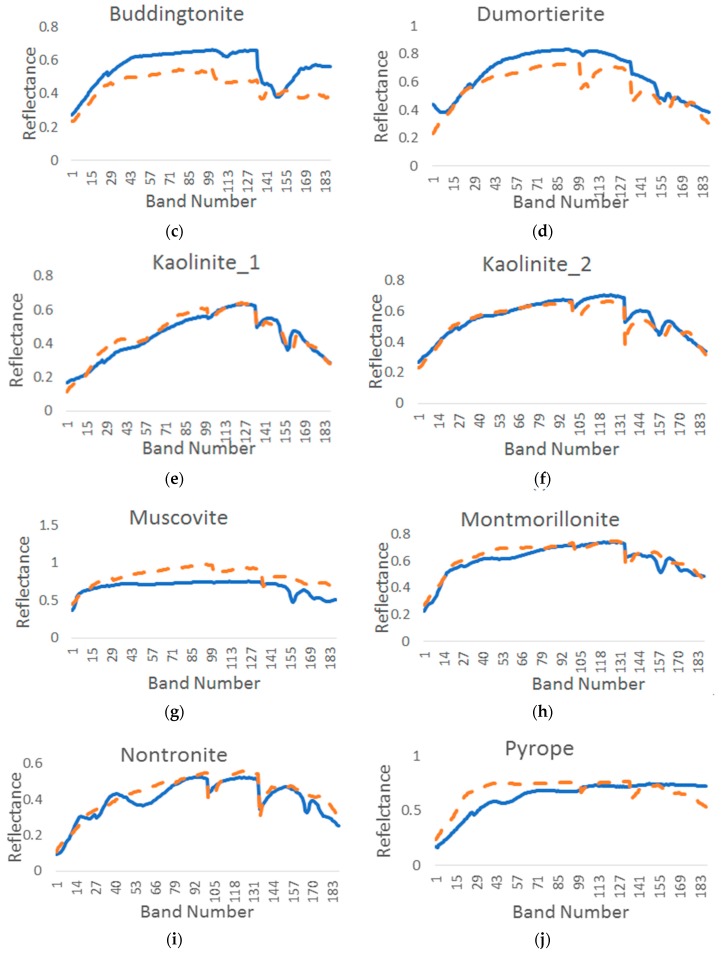

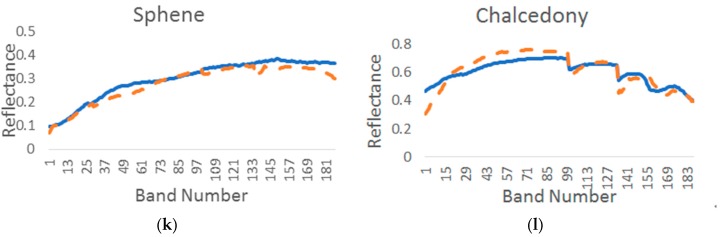
Results on the AVIRIS Cuprite image: Comparison of the USGS library spectra (solid line) with the endmember spectra extracted by PISINMF (dotted line). (**a**) Alunite; (**b**) Andradite; (**c**) Buddingtonite; (**d**) Dumortierite; (**e**) Kaolinite_1; (**f**) Kaolinite_2; (**g**) Muscovite; (**h**) Montmorillonite; (**i**) Nontronite; (**j**)Pyrope; (**k**) Sphene; (**l**) Chalcedony.

Figure 17 displays a comparison of the endmember spectra of the PISINMF algorithm with the spectral curve of the USGS library. The endmember spectral curves of the PISINMF algorithm are represented by dotted lines, and the reference endmember spectral curves of the USGS library are represented by solid lines in Figure 17. Table 10 provides SAD values for each method (PISINMF, L_1/2_-NMF, MVC-NMF, MVES), and the best result is denoted by bold font. As can be seen from Table 10, the PISINMF algorithm has the highest number of endmember minimum SAD values, and the PISINMF algorithm has the smallest endmember SAD mean value. So the PISINMF algorithm is superior to other algorithms (L_1/2_-NMF, MVC-NMF and MVES).

## 4. Discussion

In this study, we investigate the validity of preserving intrinsic structure invariant in hyperspectral unmixing to improve spectral unmixing accuracy. The experimental results of synthetic data and real data prove that the proposed PISINMF algorithm is superior to the typical algorithms (i.e., MVC-NMF, L_1/2_-NMF, MVES, VCA). However, some issues still need to be resolved or improved for further research.

First, since the true ground object distribution is unknown, the intrinsic structure of the true ground object distribution cannot be modeled. However, we can make full use of hyperspectral imagery as a priori knowledge and model its intrinsic structure. Equation (8) which utilizes spatial information and spectral information of hyperspectral image is chosen as projection equation to model the intrinsic structure of hyperspectral image. If the spatial distance between the surrounding pixel and the central pixel is farther, the weaker the relation between them is. The relation between them is mapped by Equation (8), and the projection value is small. If the surrounding pixels are closer to the central pixel, the stronger the relation between them is. The relation between them is mapped by Equation (8), and the projection value is large. The result value of Equation (8) can reflect the distance information between the surrounding pixels and the central pixel to some extent. Meanwhile, ***R*** matrix based on Equation (8) can segment the homogeneous and transition areas of hyperspectral images. Segmentation image inherits the spatial distribution characteristics of the original hyperspectral image. Moreover, compared with Equation (3), Equation (8) utilizing spectral information better reveal the real situation of the distribution of ground objects, which has been explained in the previous examples. As mentioned earlier, graph regularizer which establishes a close link between hyperspectral image and decomposed abundance matrix is introduced in PISINMF algorithm model to keep intrinsic structure invariant between hyperspectral image and decomposed abundance matrix. Synthetic hyperspectral data experiments prove that graph regularizer can promote the intrinsic structure of the estimated abundance obtained by PISINMF closer to the intrinsic structure of true abundance.

**Figure 18:**
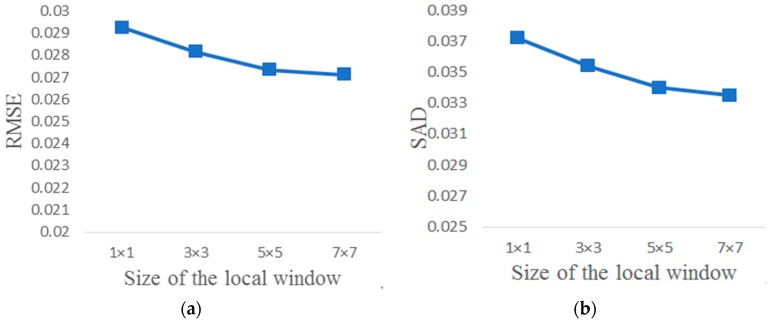
Experimental results with different sizes of the local window: (**a**) RMSE; (**b**) SAD.

Second, in the PISINMF algorithm model, how to choose the size of the local window is considered. Different sizes of local windows affect the spectral unmixing accuracy. In Figure 18, as the size of the local window becomes larger, the spectral unmixing accuracy is improved. But choosing a large size of the local window consumes more computation. Meanwhile, a large size of the local window means that the local window contains surrounding pixels that are farther away from the central pixel. According to Equation (8), if the spatial distance of the surrounding pixel from the central pixel is further, its effect on the central pixel is smaller. So there is no need to choose a large size of the local window. It is reasonable to select the size of the local window as 5 × 5 in PISINMF algorithm model.

Third, L_1/2_ regularizer as sparsity constraints has been introduced into the PISINMF algorithm model to improve the sparsity of endmember abundances. How to select the regularization parameter λ to control the impact of the sparsity constraints is an open theoretical issue. In [44], the regularization parameter λ is dependent on the sparsity criteria, which is widely adopted in other spectral unmixing methods [20]. The optimization method mentioned in [20] can only guarantee that the sparse solution converges to the local minimum. However, due to the non-convexity of the non-negative matrix function, there are multiple local minima. To avoid getting stuck in local minima, we refer to the research [31] to define the regularization parameter λ in the paper. Additionally, the experimental results show that it is superior to traditional sparsity criteria.

## 5. Conclusions

The PISINMF model, which can preserve intrinsic structure invariant in hyperspectral unmixing, is proposed in the paper. In the PISINMF model, a novel projection equation which utilizes spatial information and spectral information of hyperspectral image is adopted to model the intrinsic structure of hyperspectral image data. Compared with the heat kernel, a novel projection equation can better reveal the real situation of the distribution of ground objects. Graph regularizer establishes a close link between hyperspectral image and abundance matrix and is introduced in PISINMF algorithm model to keep intrinsic structure invariant. Compared with VCA and L_1/2_-NMF, the experiments of synthetic hyperspectral data show that the intrinsic structure of the estimated abundance obtained by PISINMF is closer to the intrinsic structure of true abundance. Besides, sparse constraints that reduces the risk of getting stuck in local minima in non-convex minimization computations is introduced into the PISINMF model, which enhances the sparsity of endmember abundances. The PISINMF model is compared with several classical hyperspectral unmixing models, including VCA, L_1/2_-NMF, MVC-NMF and MVES. The experimental results of synthetic hyperspectral data with different SNR levels, mixing coefficients and pixel numbers prove that the PISINMF algorithm model is superior to other classical methods using SAD and RMSE standards. Two real hyperspectral datasets are experimented to verify the effectiveness of the PISINMF model. Experimental results on two real hyperspectral datasets demonstrate that the PISINMF model outperforms other several classical algorithms. Specifically, the overall SAD accuracy of the PISINMF model is approximately 9–22% higher than that of the L_1/2_-NMF model.

## Figures and Tables

**Table 1:** Resulting projection values.

Projection	Equation (3)	Equation (8)
Projection value of the relation between tree1 and tree2	0.0079	0.1640
Projection value of the relation between tree1 and rock	0.0069	0.0220

**Table 2:** The error values of the intrinsic structure of estimated abundance.

	PISINMF	L_1/2_-NMF	VCA
NORMp	0.5741	0.7053	0.7120

**Table 3:** SAD comparison with different SNR

SNR	PISINMF	L_1/2-_NMF	VCA
15	0.0336	0.0370	0.0435
30	0.0328	0.0372	0.0430
45	0.0270	0.0318	0.0372
60	0.0266	0.0318	0.0412
∞	0.0292	0.0331	0.0401

**Table 4:** RMSE comparison with different SNR.

SNR	PISINMF	L_1/2_-NMF	VCA
15	0.0895	0.0941	0.1015
30	0.0682	0.0762	0.0855
45	0.0569	0.0674	0.0740
60	0.0654	0.0694	0.0730
∞	0.0635	0.0705	0.0729

**Table 5:** SAD comparison with different abundance mixing coefficients.

Mixing Coefficients	PISINMF	L_1/2_-NMF	VCA
0.6	0.0630	0.0700	0.0740
0.7	0.0305	0.0353	0.0557
0.8	0.0403	0.0440	0.0557
0.9	0.0119	0.0186	0.0205

**Table 6:** RMSE comparison with different abundance mixing coefficients.

Mixing Coefficients	PISINMF	L_1/2_-NMF	VCA
0.6	0.2416	0.2645	0.2681
0.7	0.1997	0.2199	0.2228
0.8	0.1853	0.2033	0.2105
0.9	0.1768	0.1859	0.1919

**Table 7:** SAD comparison with different numbers of pixels in the scene.

Number of Pixels	PISINMF	L_1/2_-NMF	VCA
900	0.0288	0.0314	0.0360
2500	0.0281	0.0319	0.0370
4900	0.0278	0.0321	0.0388
8100	0.0297	0.0334	0.0395

**Table 8:** RMSE comparison with different numbers of pixels in the scene.

Number of Pixels	PISINMF	L_1/2_-NMF	VCA
900	0.0549	0.0656	0.0678
2500	0.0558	0.0649	0.0682
4900	0.0569	0.0652	0.0678
8100	0.0577	0.0659	0.0687

**Table 9:** SAD results on the Samson data.

	PISINMF	L_1/2_-NMF	MVC-NMF	MVES
Water	**0.0798**	0.0835	0.1365	0.1895
Tree	**0.0612**	0.0728	0.0968	0.0653
Rock	**0.0123**	0.0168	0.0298	0.0438
AVERAGE	**0.0511**	0.0577	0.0877	0.0995

**Table 10:** SAD results on the AVIRIS Cuprite data.

	PISINMF	L_1/2_-NMF	MVC-NMF	MVES
Alunite	0.1433	0.2305	0.2380	**0.1302**
Andradite	0.1082	**0.0673**	0.0750	0.1444
Buddingtonite	**0.0873**	0.1117	0.1015	0.2638
Dumortierite	0.0797	0.1230	**0.0757**	0.1288
Kaolinite#1	**0.0693**	0.1025	0.1360	0.1304
Kaolinite#2	**0.0564**	0.0968	0.1374	0.1222
Muscovite	0.0878	**0.0848**	0.1434	0.2132
Montmorillonite	**0.0553**	0.0635	0.0601	0.1565
Nontronite	**0.0809**	0.0824	0.0864	0.2949
Pyrope	0.1548	**0.0809**	0.1267	0.1127
Sphene	**0.0529**	0.1168	0.2490	0.1423
Chalcedony	**0.0804**	0.1315	0.1045	0.1502
AVERAGE	**0.0880**	0.1132	0.1314	0.1697
